# Draft Genome Sequence of *Mycobacterium europaeum* Strain CSUR P1344

**DOI:** 10.1128/genomeA.00816-15

**Published:** 2015-07-23

**Authors:** Michael Phelippeau, Olivier Croce, Catherine Robert, Didier Raoult, Michel Drancourt

**Affiliations:** Aix Marseille Université, URMITE, UMR CNRS 7278, IRD 198, INSERM 1095, Faculté de Médecine, Marseille, France

## Abstract

We report the draft genome sequence of *Mycobacterium europaeum* strain CSUR P1344, a slowly growing mycobacterium of the *Mycobacterium simiae* complex and opportunistic respiratory tract colonizer and pathogen. This genome of 6,152,523 bp exhibits a 68.18% G+C content, encoding 5,814 predicted proteins and 74 RNAs.

## GENOME ANNOUNCEMENT

*Mycobacterium europaeum* was described in 2011 on the basis of five isolates made from the respiratory tract and lymph nodes of patients in Italy, Greece, and Sweden (1). Two further cases were reported in the respiratory tract of Iranian patients (2). Recently, we acquired one *M. europaeum* isolate from the respiratory tract of a patient with a history of HIV-HCV coinfection and acute flu (3), and one further isolate from the sinus surgical drainage of a patient with sinus abscess. Both isolates were identified on the basis of a 100% similarity with the *rpoB* partial sequence (1, 4). To date, only 20 partial sequences of the 16S rRNA, 16S–23S rRNA intergenic spacers—*hsp65*, *tuf*, *sodA*, *hsp65*, *groEL2*, and *rpoB*—are available for *M. europaeum*. We thus performed whole-genome sequencing of the *M. europaeum* CSUR P1344 isolate (3) in order to increase knowledge and gain insights into this organism, which is potentially involved in the contamination and/or infection of airways.

Genomic DNA was isolated from *M. europaeum* strain CSUR P1344 and grown onto Middlebrook 7H10 solid medium (Becton, Dickinson, Le Pont-de-Claix, France) at 37°C in an atmosphere enriched with 5% CO_2_. It was sequenced by parallel paired-end and mate pair high-throughput MiSeq technology (Illumina Inc, San Diego, CA, USA). Starting from 1.5 µg genomic (gDNA) fragmented with an optimal size at 6 kb, the mate pair Nextera library was constructed with an input of 1 ng of gDNA. Both libraries were sequenced on MiSeq in 2 × 251 bp, and final sequencing produced a total of 1,047,703 reads.

The whole set of reads was trimmed using Trimmomatic (5) and then assembled through the assembler software Spades (6, 7). Contigs were combined together by SSPACE (8), Opera (9) helped by GapFiller (10), and homemade tools in Python to refine the set. Finally, the draft genome of *M. europaeum* CSUR P1344 consists of 13 contigs without gaps, containing 6,152,523 bp. The G+C content of this genome is 68.18%.

Noncoding genes and miscellaneous features were predicted using RNAmmer (11), ARAGORN (12), Rfam (13), Pfam (14), and Infernal (15). Coding DNA sequences (CDSs) were predicted using Prodigal (16), and functional annotation was achieved using BLAST+ (17) and HMMER3 (18) against the UniProtKB database (19). The genome was shown to encode at least 74 predicted RNAs, including 3 rRNAs, 50 tRNAs, 1 tmRNA, and 20 miscellaneous RNAs. A total of 5,814 identified genes yielded a coding capacity of 5,560,314 bp (coding percentage: 90.37%). Among these genes, 295 (5.07%) were found to be putative proteins and 1,061 (18.25%) were assigned as hypothetical proteins. Moreover, 4,005 genes matched at least one sequence in the Clusters of Orthologous Groups (COGs) database (20, 21) with BLASTp default parameters.

### Nucleotide sequence accession numbers.

The *M. europaeum* CSUR P1344 strain genome sequence has been deposited with its annotations at EMBL under the accession numbers CTEC01000001 to CTEC01000013.
